# Time to first childbirth and its predictors among reproductive-age women in Ethiopia: survival analysis of recent evidence from the EDHS 2019

**DOI:** 10.3389/frph.2023.1165204

**Published:** 2023-07-14

**Authors:** Tegene Atamenta Kitaw, Ribka Nigatu Haile

**Affiliations:** School of Nursing, College of Health Science, Woldia University, Woldia, Ethiopia

**Keywords:** childbirth, determinants, Ethiopia, reproductive age, survival analysis, time to first

## Abstract

**Background:**

Being a mother for the first time is the most significant event in a woman's life. “Age at first birth” refers to a mother's age in years when she gives birth to her first child. The age of first childbirth has physical, economic, and social implications. However, little is known about this issue in Ethiopia. Thus, this study sought to determine the time to first childbirth and its predictors at a national level.

**Methods:**

Data were extracted from the 2019 Ethiopia Demographic and Health Survey using STATA version 17 software. A total of 8,885 weighted reproductive-age women (15–49 years) were included in this study. A Kaplan–Meier survivor curve was generated to estimate the time of first childbirth. A log-rank test was used to compare the difference in survival curves. Akaike information criteria and Bayesian information criteria were calculated to select the appropriate survival model for the data. The Weibull accelerated failure time model with no frailty distribution was used to identify significant predictors.

**Results:**

The overall median survival time to first childbirth was 18 years. The significant predictors of time to first childbirth were the educational level of the mother [primary education (ϕ = 1.036, 95% CI: 1.011, 1.063), secondary and above education (ϕ = 1.154, 95% CI: 1.118, 1.191)], knowledge of any contraceptive method [know at least one (ϕ = 1.051, 95% CI: 1.006, 1.101)], and media exposure (ϕ = 1.048, 95% CI: 1.011, 1.086).

**Conclusion:**

The median survival time to first childbirth was 18 years, which is lower than the optimal age for first childbirth (late 20 s and early 30 s). The timing of first childbirth in Ethiopia is mainly influenced by the educational level of women, knowledge of contraceptive methods, and exposure to media. Thus, exposing women to educational materials and other awareness-creation campaigns regarding the consequences of early first childbirth and strategies to improve women's knowledge of contraceptive methods is highly recommended.

## Background

Age at first birth refers to the woman's age in years when she gives birth to her first child (1). Childbirth is the most significant event in a woman's life. It is an intense event that is physically, psychologically, socially, and culturally significant (2). The impact could be positive, both in the short and long term; however, sometimes, it could result in negative and traumatizing events (3).

Globally, nearly 16 million girls aged between 15 and 19 give birth to their first child each year (4). In developing countries, approximately one-third of women begin having children at 19 or younger, and nearly half of the first births in adolescence are from girls aged 17 or younger. Half of the girls who give birth between the ages of 15 and 17 have a second birth in adolescence, and 11 percent of girls with two births have the third birth in adolescence (5). Early pregnancy and motherhood are common in east African countries, ranging from 18% in Kenya and 29% in Malawi (6).

The socioeconomic implications of youth pregnancy and early childbearing are significant in developing nations and are linked with maternal mortality, low birth weight, poor school achievement and productivity, and, subsequently, intergenerational poverty transmission (7, 8). As the age at first birth decreases, the likelihood of maternal mortality rises. Girls who give birth under 15 and between 15 and 19 years of age are five and two times more at risk of mortality related to pregnancy and childbirth consequences, respectively, than women aged 20–24 at first birth (9, 10). Additionally, pregnancy-related consequences, such as eclampsia, obstetric fistula, and systemic infection, are linked to early age at first birth (11). Later in women's lives, early age birth is also linked to an increased risk of diabetes mellitus, hypertension, lung disease, and poor physical performance (12). In addition to having an adverse effect on the mother's health, it also has negative repercussions on their education, work prospects, and opportunities. Moreover, it is related to societal consequences such as violence, rejection, and shame (13). Additionally, early age at first birth significantly affects a country's population growth, particularly in countries where modern contraception is underutilized (11, 14). Under 5 morbidity is also greater among children born to mothers under 20 years of age (15). Additionally, the risk of newborn mortality is considerably higher for infants whose mothers are under 16 years of age (16). However, bearing a first child at an advanced age is associated with a higher risk of miscarriage, multiple pregnancies, diabetes, chromosomal abnormities, and maternal mortality (10, 17).

The age at first birth varies across different regions. Among European Union (EU) countries the mean age of women at first childbirth ranges from 26.3 in Bulgaria to 31.3 in Italy (18). The median ages at first birth in East Asia and the Pacific (19), Bangladesh (20), Nigeria and Ghana (21, 22), Sub-Saharan Africa (23), and Uganda are 20.2, 16.34, 20, 19, and 19.2 years, respectively (24). In Ethiopia, 13% of women aged 15–19 have begun childbearing and 2% are pregnant with their first child. One in two women aged 25–49 give birth for the first time before age of 20 years (25).

Lower level educational status (26–28), rural residency (21), poor wealth index (29), unemployment (14), husband's education and occupation (30), early age at first sexual intercourse (28, 31), younger age at first marriage (32, 33), peer pressure (34), and smoking status (35) were identified as predictors for early age at first childbirth in several studies.

Delaying pregnancy and childbearing in adolescent women may result in higher academic achievement, ensuring women's economic independence and a better life. Even though promising progress has been made so far regarding age at first birth, early childbearing remains a great challenge in Ethiopia. In 2021, Ethiopia launched a new national adolescent and youth health strategy (2021–2025) with the aim of reducing early age at first pregnancy from 13% to 7% and increasing the median age at first marriage from 17 to 18, thereby reducing early age at first birth (36). Understanding the current nationwide status of age at first birth will play an enormous role in achieving the above-stated goal.

A few related studies have been undertaken so far in Ethiopia. Most studies focused on only teenage and/or adolescent childbearing (37, 38). Furthermore, studies were limited to specific districts/areas (39). Survival analysis of time to first birth among reproductive-age women will have a paramount role in subduing those limitations and further estimating the significant impact of predictor variables at a national level. The age at which childbearing first commences will have a crucial role in estimating the overall fertility level at the country level. Furthermore, this study uses recent Ethiopia Demographic and Health Survey (EDHS, 2019) data, which is crucial in providing up-to-date information on national improvements regarding early childbearing.

## Methods

### Study setting, study period, and data source

Based on the latest census figures and projections from trading economics, the total population in Ethiopia was estimated at 115.0 million people in 2020 (40). The final report of the EDHS 2019 contained detailed information at a national level from the nine regional states and two city administrations of Ethiopia. The administration levels went from regions to zones and through woreda. A survival analysis was conducted among reproductive-age group (15–49) women in Ethiopia using the EDHS data. The EDHS was implemented by the Ethiopian Public Health Institute (EPHI) in collaboration with the Central Statistical Agency (CSA) and the Federal Ministry of Health (FMoH). The target populations were women aged 15–49 and men aged 15–59 in selected households across Ethiopia. The EDHS contains information on the background characteristics of the respondents, maternal health care, fertility, marriage and sexual activity, child feeding practices, nutritional status of women and children, and adult and childhood mortality. Data collection was carried out from March to June 2019 (41).

### Data extraction and population

We received a letter permitting us to acquire the EDHS 2019 data from the DHS program after making a reasonable request. Data extraction was carried out to select reproductive-age women. For this study, a weighted sample of 8,885 reproductive-age women was drawn. The data extraction period was from October 1, 2022 to October 15, 2022. All reproductive-age women (15–49 years) in Ethiopia were the source population, whereas all reproductive-age women (15–49 years) in Ethiopia in the selected enumeration area were the study population.

### Sampling methods

**Figure 1:**
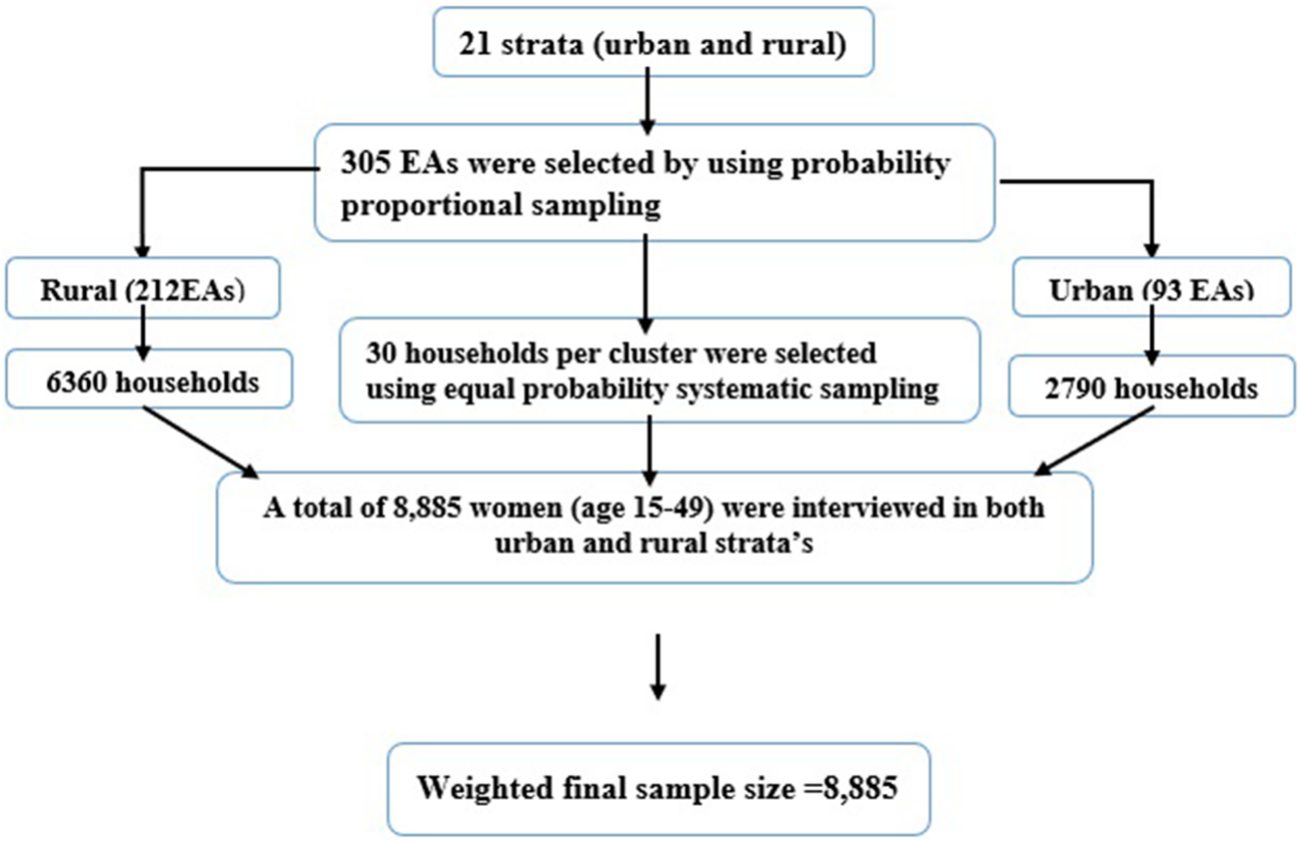
Schematic representation of the sampling procedures in the study of time to first childbirth and its predictors among reproductive age women in Ethiopia (EDHS, 2019). EAs; enumeration areas.

The 2019 EDHS sample was stratified and selected in two stages. Each region was stratified into urban and rural areas, yielding 21 sampling strata. In the first stage, 305 enumeration areas (EAs) (93 in urban areas and 212 in rural areas) were selected with a probability proportional to EA size. In the second stage, a fixed number of 30 households per cluster were selected with an equal probability systematic selection from the newly created household listing. Sample allocation was carried out to ensure that survey precision was comparable among regions. A total of 25 EAs were selected from eight regions (including two city administrations), and 35 EAs were selected from the three largest regions: Amhara, Oromia, and the Southern Nations, Nationalities, and Peoples' Region (SNNPR). The detailed sampling procedure is accessible in the EDHS 2019 report (41). In this study, a total of 8,885 weighted reproductive-age women (15–49 years) were included. The highlighted sampling procedure for this study is indicated in the figure below (Figure 1).

### Inclusion and exclusion criteria

All reproductive-age women (15–49 years) in the selected enumeration area were included in the study, whereas women declared infecund were excluded.

### Study variables

The dependent variable is the time (in years) at first childbirth. This study considered different explanatory variables to determine predictors of time to first childbirth (Table 1).

**Table 1:** List of explanatory variables for the assessment of time to first childbirth in Ethiopia.

	Description
Woman's age	Categorized as 15–24, 25–34, and 35–49
Residence	Categorized as urban or rural
Mother educational level	Categorized as no education, primary, secondary, or higher
Wealth index	Categorized as poor, middle, or rich
Marital status	Not married or married
Sex of household head	Categorized as male or female
Household size	Categorized as 1–4, 5–9, and 10 and more
Contraceptive use and intension	Does not intend, using traditional method and using modern method
Knowledge of any contraceptive method	Knows no method or knows at least one method
Media exposure (television)	Yes or no
Media exposure (radio)	Yes or no

### Operational definition

Event: mothers who first gave birth until the 2019 EDHS data collection end date. Censored: women who did not first give birth until the 2019 EDHS data collection date. Time to first birth: age of the mother in years when she first gave birth (42).

### Data processing and analysis

STATA version 17 software was used to extract data from the EDHS 2019 individual (women) record folder. The data were coded, cleaned, and edited. Listing and sorting were carried out to find any missing values. Descriptive statistics were analyzed and presented in terms of frequency and percentage. Age at first birth is calculated as the age difference between the mother and her oldest child. A Kaplan–Meier survivor curve was used to determine the time (years) of first childbirth. A log-rank test was computed to compare the difference in survival curves between categories of variables. A Shenfield residual was fitted and computed to test the assumption of the Cox proportional hazard. Akaike information criteria (AIC) and Bayesian information criteria (BIC) were calculated to select the appropriate survival model for the data. Multicollinearity was checked before running the selected survival model. A VIF above 4 or tolerance below 0.25 indicated that multicollinearity might exist (43). In this study, the maximum VIF was 2.30 with a mean VIF of 1.54 and the minimum tolerance value was 0.43. Thus, there was no multicollinearity between covariates. Variables with a *p* ≤ 0.25 in the bivariate analysis were fitted and included in the multivariable Weibull accelerated failure time (AFT) model. In the multivariable analysis, variables with a *p* ≤ 0.05 were considered statically significant.

### Rationale for using the survival analysis

Survival analysis is a statistical method for analyzing data in which the outcome variable of interest is the time until an event occurs. The outcome of interest in this study was the time to first childbirth, which is time to event data and do not merely depend on whether the event occurred or not but also the time at which the event occurred. Thus, it is best suited to use a survival analysis model. Furthermore, the outcome of interest contained both an event and a time. Thus, linear and logistic regressions were not appropriate. Additionally, those regression models are not well enough equipped to handle censoring events.

## Results

### Socio-demographic and reproductive health-related characteristics

A total of 8,885 weighted reproductive-age women were included for the examination on the time to first childbirth in Ethiopia; 5,855 (65.9%) of women gave birth to at least one child (event). Weighted frequency analysis showed that 6,024 (67.8%) of the respondents resided in rural areas. Regarding educational status, 3, 589 (40.4%) of the respondents had no formal education. A total of 3,052 (34.4%) reproductive age women were in the poor household wealth index category. Concerning knowledge of any contraceptive method, 424 (4.8%) of the women did not know of any contraceptive method (Table 2).

**Table 2:** Socio-demographic and reproductive health-related characteristics of women in Ethiopia (EDHS, 2019).

Variable	Categories	Weighted frequency	First childbirth status	
			Censored (%)	Event (%)
Age	15–24	3,691	2,635 (29.5%)	1,055 (11.9%)
	25–34	2,827	325 (3.7%)	2,502 (28.2%)
	35–49	2,367	69 (0.8%)	2,298 (25.9%)
Residence	Urban	2,861	1,188 (13.4%)	1,673 (18.8%)
	Rural	6,024	1,842 (20.7%)	4,182 (47.1%)
Mother education level	No education	3,589	365 (4.1%)	3,224 (36.3%)
	Primary	3,701	1,700 (19.1%)	2,001 (22.5%)
	Secondary and above	1,595	964 (10.9%)	630 (7.1%)
Wealth index	Poor	3,052	856 (9.6%)	2,196 (24.75%)
	Middle	1,671	526 (5.9%)	1,145 (12.9%)
	Rich	4,162	1,648 (18.5%)	2,514 (28.3%)
Marital status	Not married	2,325	2,274 (25.6%)	51 (0.6%)
	Married	6,560	755 (8.5%)	5,804 (65.3%)
Household size	1–4	3,175	1,134 (12.8%)	2,040 (23.0%)
	5–9	5,180	1,662 (18.7%)	3,519 (39.3%)
	10 and more	530	234 (2.6%)	296 (3.3%)
Contraceptive use and intention	User	2,556	253 (2.9%)	2,303 (25.9%)
	Nonuser	6,329	2,777 (31.2%)	3,553 (40.0%)
Knowledge of any contraceptive method	Knows no method	424	212 (2.4%)	212 (2.4%)
	Knows at least one method	8,461	2,818 (31.7%)	5,643 (63.5%)
Media exposure (television)	No	7,027	2,274 (26.0%)	4,753 (54.4%)
	Yes	1,716	668 (7.6%)	1,048 (12.0%)
Media exposure (radio)	No	6,219	2,064 (23.6%)	4,155 (47.5%)
	Yes	2,523	877 (10.0%)	1,646 (18.8%)

### Survival time of first childbirth

**Figure 2:**
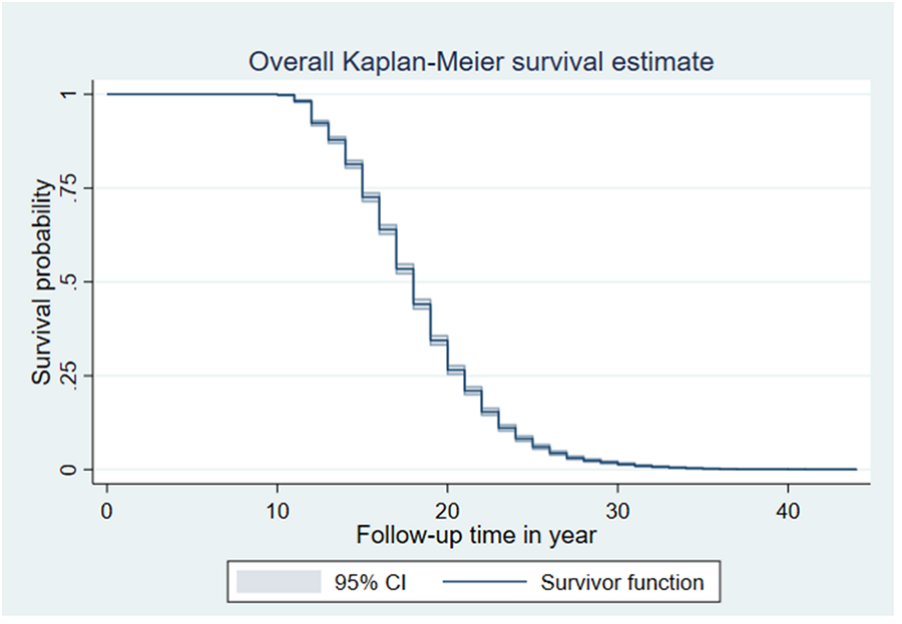
Overall Kaplan–Meier failure curve of time to first childbirth in Ethiopia (EDHS 2019).

The overall median survival time to first childbirth was 18 years. The total follow-up time contributed by all study participants was 107,124 person years. The survival probability of time to first childbirth beyond 14, 16, 18, and 20 years was 87.8%, 72.6%, 53.5%, and 34.4%, respectively (Figure 2).

The median survival time differed among the participant characteristics. The median survival time was 19 years for women who resided in an urban area and 17 years for women from a rural area. By educational status, the median survival time was 17 years for women with no formal education and 20 years for those with secondary and above education. Regarding the wealth index, the median survival times for poor, middle, and rich were 17, 18, and 19 years, respectively.

### Comparisons of survival functions of different categorical variables

A Kaplan–Meier survival curve and log-rank test were computed to compare and estimate the survivor function among different characteristics of the respondent. In the Kaplan–Meier survival curve, one survivorship function curve located under another implies that the lower curve group has a lower survival status than the upper curve group. Furthermore, the difference is explained statistically by the log-rank test.

**Figure 3:**
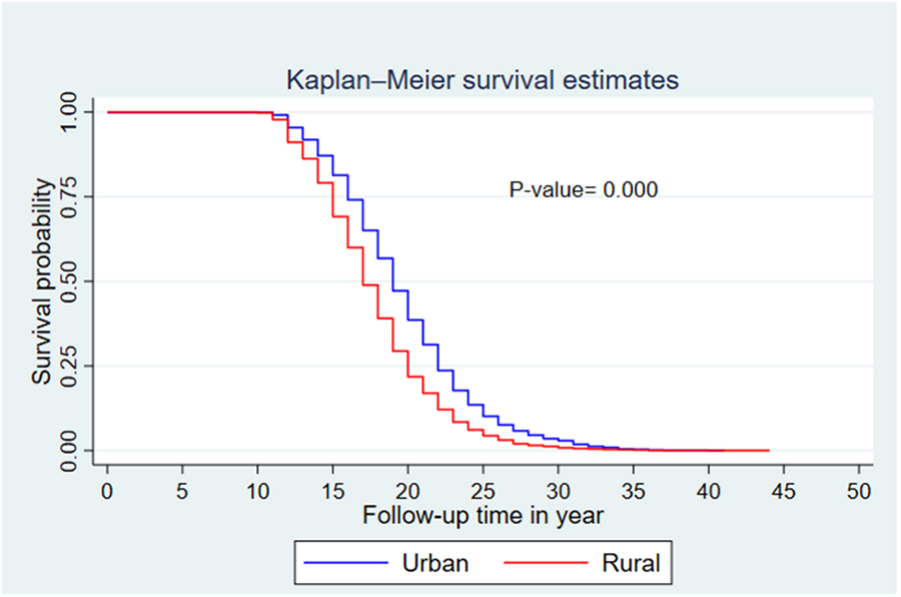
Kaplan–Meier survival curves and log-rank tests by residence status (EDHS 2019).

**Figure 4:**
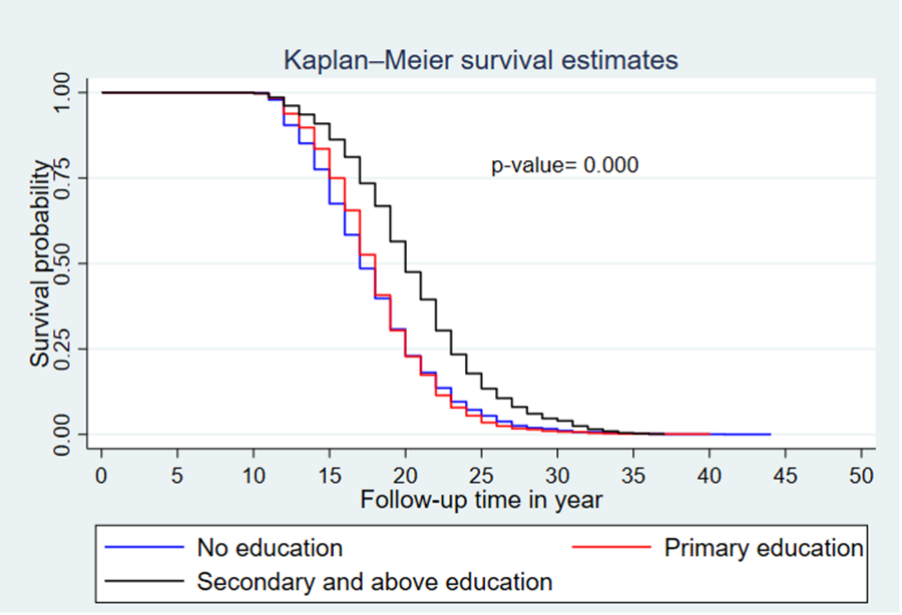
Kaplan–Meier survival curves and log-rank tests by women's educational level (EDHS 2019).

**Figure 5:**
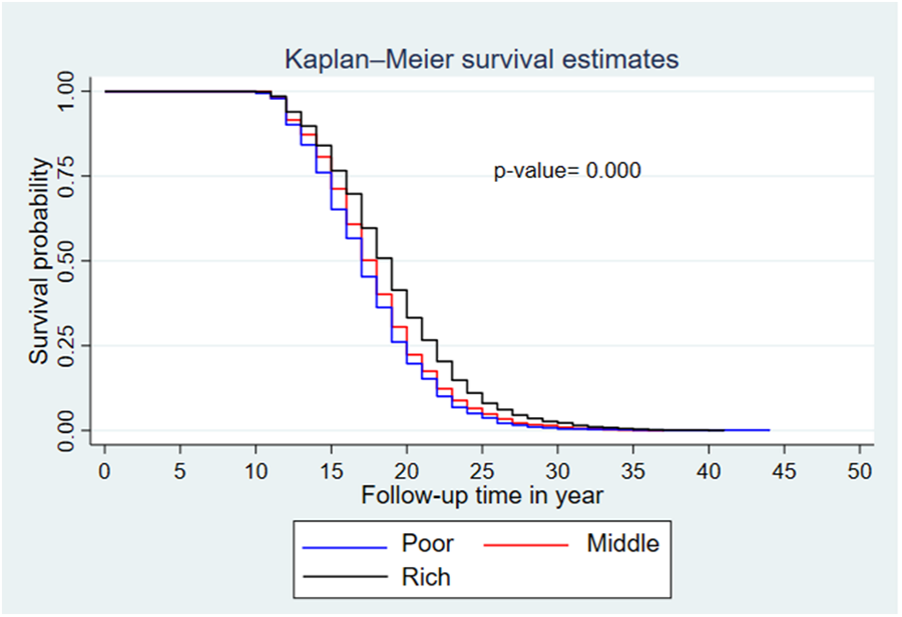
Kaplan–Meier survival curves and log-rank tests by wealth index level (EDHS 2019).

Generally, the Kaplan–Meier survival curve shows that women who reside in a rural area, have no formal education, and have lower wealth index categories had their first child earlier than the reverse group. Furthermore, the log-rank test *p*-value showed that there was a significant difference in survival experience among covariates of residence (*p* < 0.001) (Figure 3), women's educational level (*p* < 0.001) (Figure 4), and wealth index categories (*p* < 0.001) (Figure 5).

## Model selection

### Proportional hazard assumption test by schoenfeld residual

The rho statistic *p*-value of all covariates and the global test *p*-value in the Schoenfeld residual was below 0.05. Therefore, the proportional hazard assumption was not fulfilled. Thus, the accelerated failure time model was considered.

### Accelerated failure time model test

The model with the smallest *AIC* and *BIC* values was considered to be the best survival model for the given data. The Weibull accelerated failure time (AFT) model with no frailty was found to have the smallest *AIC* and *BIC* values (299.23 and 225.82). Thus, the Weibull accelerated failure time (AFT) model with no frailty was the best model for the data to describe the status of women towards time to first childbirth and its predictors (Table 3).

**Table 3:** Comparison of akaike information criteria and Bayesian information criteria among different accelerated failure time model and frailty distributions.

Information criteria	Models	Frailty distributions		
		No frailty	Gamma frailty	Inverse Gaussian frailty
AIC	EXPONENTIAL	11,996	11,998	11,998
	WEIBULL	**299** **.** **23**	966.86	808.86
	LOG-LOGISTIC	946.89	944.89	944.89
	LOG-NORMAL	1,054.49	1,052.49	1,052.49
	GENERALIZED GAMMA	1,052.69	910.81	967.50
BIC	EXPONENTIAL	12,062.73	12,071.4	12,071.4
	WEIBULL	**225** **.** **82**	886.78	728.78
	LOG-LOGISTIC	873.49	864.81	864.81
	LOG-NORMAL	981.09	972.41	972.41
	GENERALIZED GAMMA	972.61	824.06	914.12

Bold value indicates the lowest Akaike information criteria and Bayesian information criteria value.

### Predictors of time to first childbirth

In the bivariable Weibull AFT model, age, residence, mother education level, wealth index, sex of household head, knowledge of any contraceptive method, media exposure (television), and media exposure (radio) were found to be significant, with a *p* ≤ 0.25. Additionally, in this model, mother educational level, knowledge of any contraceptive method, and media exposure (television) were predictors of time to first childbirth.

The acceleration factor for time to first childbirth among mothers who had secondary and above education level was 1.154 (*ϕ* = 1.154, 95% CI: 1.118, 1.191) compared with an uneducated mother. Additionally, the acceleration factor for time to first childbirth in mothers with only primary education was 1.036 (*ϕ* = 1.036. 95% CI: 1.011, 1.063) compared with the reference group (no education). This shows that uneducated women gave birth to their first child earlier than educated mothers.

Regarding knowledge towards contraceptive methods, the acceleration factor of time to first birth for women who know of at least one contraceptive method was 1.051 (*ϕ *= 1.051, 95% CI: 1.006, 1.101). This implies that women with knowledge of at least one contraceptive method have a delayed age at first birth compared to women without knowledge of contraceptive methods. In another way, women without knowledge of any contraceptive methods had their first child earlier than their counterparts. Women with media exposure (television) have a 1.048 times acceleration factor for time to first childbirth compared with the reverse group. (*ϕ *= 1.048, 95% CI: 1.011, 1.086) (Table 4).

**Table 4:** Bivariable and multivariable weibull accelerated failure time (AFT) model analysis for predictors of time to first childbirth in Ethiopia **[**EDHS 2019 **(***n* = 8,885**)**].

Variable	Categories	Coef.	Acceleration factor (ϕ)	95% CI for ϕ	*p*-value
Residence	Urban	1	1	1	1
	Rural	−0.0097	0.990	(0.953, 1.029)	0.618
Mother educational level	No education	1	1	1	1
	Primary	0.0358	1.036	(1.011, 1.063)	**0**.**006**
	Secondary and above	0.1432	1.154	(1.118, 1.191)	**0**.**000**
Wealth index	Poor	1	1	1	1
	Middle	−0.0161	0.984	(0.957, 1.012)	0.264
	Rich	−0.0231	0.977	(0.948, 1.007)	0.136
Sex of household head	Male	1	1	1	1
	Female	0.0209	1.021	(0.992, 1.051)	0.153
Knowledge of any contraceptive method	Knows no method	1	1	1	**1**
	knows at least one method	0.0509	1.052	(1.006, 1.101)	**0**.**028**
Media exposure (television)	No	1	1	1	**1**
	Yes	0.0467	1.048	(1.011, 1.086)	**0**.**011**
Media exposure (radio)	No	1	1	1	1
	Yes	−0.0152	0.985	(0.957, 1.013)	0.295

Bold value indicates variables having a *p*-value of ≤0.05.

## Discussion

Time to first childbirth and its predictors were determined by using data from the recent 2019 Ethiopian Demographic Health Survey. Mother education level, knowledge of any contraceptive method, and media exposure (television) were found to be predictors of time to first childbirth.

The overall median survival time (age) to first childbirth was 18 years. This finding is in line with a study carried out in Gambia (18 years) (44). This result is lower than the that from a study undertaken in several regions, such as the USA (26.9 years) (45), Ghana (19.91 years) (22), Uganda (19.2 years) (24), Nigeria (20 years) (33), and Kenya (20.3 years) (46). The awareness of women about the consequence of having babies at an early age and contraceptive access and utilization plays a paramount role in delaying age at first childbirth in developed countries (47). In developing countries like Ethiopia, women's autonomy regarding reproductive health decisions is low. This might lead to poor reproductive health care seeking behaviors, such as low contraceptive utilization, thereby resulting in early first childbearing (48, 49). In addition, the variation could be attributed to different factors such as age at first marriage, age at first sexual experience, and contraceptive usage, which were listed as factors that shortened the survival time of onset of first childbirth in different studies (22, 28, 50). For instance, the median ages at first marriage in Ethiopia and Kenya were 17.2 years (51) and 19 years (52), respectively. In addition, contraceptive use is quite different; the prevalence of contraceptive use in Kenya is 39% (53), whereas as in Ethiopia it is 20.42% (54).

On the contrary, the finding of this study is higher than the study conducted in Bangladesh (17.92 years) (55). This variation could be attributed to differences in religious beliefs. In Bangladesh, most of the population has a Muslim affiliation. A Muslim religious affiliation is closely linked with the early age of the mother at first birth (14). In addition, in Bangladesh, many people are unaware of the consequences of early marriage and early pregnancy (56).

This study revealed that women's educational level is a predictor of time to first childbirth. Women who attend primary, secondary, and higher education delayed their first childbirth more than those who did not have any education. This finding is consistent with a study carried out in Kenya (57), which concluded that the probability of giving birth at an earlier age decreases as the educational level increases. Similarly, a study carried out in Ghana revealed that the higher the woman's educational level, the longer the waiting time for her first birth: 73% of postsecondary level women had yet to give birth before the age of 30 (22). Furthermore, a study conducted in Bangladesh also found pretty consistent findings that women with higher levels of education have a lower risk of having an early first birth (42).

The possible explanation might be that uneducated women might not know the optimal age at which giving childbirth has the minimum risk. Education level and contraceptive utilization is directly proportional. The higher the education level, the higher the use of contraception (58). Thus, the low contraceptive use of uneducated mothers leads to early first childbirth. Therefore, enrolling women in at least primary education will reduce the incidence of early first childbirth and the related consequences.

This finding indicates a positive interaction between women's knowledge of contraceptive methods and delaying the time of first childbirth. This finding is supported by a study carried out in East Asia and the Pacific (19) that found that there is a high incidence of early first childbirth among women without any knowledge of contraceptive methods. Likewise, a study conducted in Tanzania (59) also revealed that contraceptive knowledge is considerably high among women who delay their first childbirth.

This might be because the likelihood of contraceptive use was high among women who know about contraceptives (60). Women might delay early-age maternity as long as she uses contraceptive methods. Thus, policymakers should emphasize improving women's knowledge of different types of contraceptives by providing access to information about contraceptives through healthcare providers, online resources, and community organizations. Furthermore, it is recommended that open dialogue about contraception is encouraged between women and their partners, family members, and healthcare providers. Thus, increasing women's knowledge of contraceptive methods can delay early maternity, preventing unintended adolescent pregnancy and early pregnancy-related mortality and morbidity.

Media exposure has also been found to be a predictor of time to first birth. Women who have access to media are less likely to give birth to their first child at an early age than those who did not have access. This result is in agreement with a study carried out in Bangladesh (61) that indicated that women exposed to media on a regular basis were less likely to give birth to their first child at an early age than those who are not exposed (44.8% vs. 70.2%).

This finding points to the fact that women who do not have access to media might be unaware of the complications associated with early age at first childbirth. In addition, maternal healthcare utilization, including family planning services, is significantly higher among women exposed to mass media, which delays early maternity (62). Furthermore, those who were not exposed to media were more likely to get married earlier and have earlier sexual experiences, resulting in early first childbirth. Thus, advertisements and educational programs through mass media targeting the consequences of early first childbirth on maternal and child health are highly recommended.

The strength of this study is that it uses nationally representative data and can be generalizable to all Ethiopian reproductive-age women. Owing to the self-reported nature of the data, there might be recall bias. As the data source is secondary, it is difficult to quantify other potential predictors of time to first childbirth. A lack of trend analysis is also a limitation of this study.

## Conclusion

In this study, the median survival time to first childbirth was 18 years, which is lower than the optimal age for first childbirth, between the late 20 s and 30 s (63, 64). This is the ideal age for education. In addition to having implications on her social, physical, and mental health, being a mother at this age may prevent the woman from attending school. The timing of first childbirth in Ethiopia is mainly influenced by women's level of educational, knowledge of contraceptive methods, and access to media. Interventions could involve raising women's educational levels by exposing them to educational materials and other awareness-creation efforts regarding the consequences of early first childbirth. In addition, expanding adolescent and youth-friendly services in the country might increase women's knowledge of contraceptive methods. Furthermore, including sexual and reproductive health education programs in the educational curriculum also contributes to reducing early marriage and sexual initiation, thereby decreasing early first childbirth. Furthermore, policymakers and other non-governmental organizations should continuously invest resources in transmitting messages through mass media (television), such as advertisements and other programs, regarding the impact of early childbirth. In conclusion, a prospective follow-up study that includes other potential predictors is recommended.

## Data Availability

The raw data supporting the conclusions of this article will be made available by the authors, without undue reservation.
