# Supplementary material for: Time to first childbirth and its predictors among reproductive-age women in Ethiopia: survival analysis of recent evidence from the EDHS 2019
Source: Front Reprod Health. 2023 Jul 14;5:1165204. doi: 10.3389/frph.2023.1165204 (PMC10382129; doi:10.3389/frph.2023.1165204)
Supplement: Supplementary file 1 [file Table1.pdf]

VIF and tolerance test to check the existence of multicollinearity between covariates

Variable	VIF	1/VIF
Residence	2.30	0.4347
Mother Educational level	1.72	0.5813
Knowledge of any contraceptive method	1.66	0.6024
Media exposure (Television)	1.59	0.6289
Wealth index	1.51	0.6622
Marital status	1.36	0.7352
Household size	1.10	0.9090
Media exposure (Radio)	1.09	0.9174
Mean VIF	1.54	

Variance inflation factor (VIF) and tolerance were computed to check the existence of multicollinearity prior to running multivariable Weibull accelerated failure time (AFT) model. A VIF above 4 or tolerance below 0.25 indicated that multicollinearity might exist (42). In this study, the maximum VIF was 2.30 with mean VIF of 1.54 and the minimum tolerance value is 0.43. . Thus, there is no multicollinearity between covariates.

.
